# Grip Strength, Cognition, and Fall risk in Mexican American Older Adults: Sex differences

**DOI:** 10.1093/geroni/igaf122.2633

**Published:** 2025-12-31

**Authors:** Winrose Windsor, Soham AlSnih

**Affiliations:** UTMB Health, The University of Texas Medical Branch, Galveston, Texas, United States; The University of Texas Medical Branch at Galveston, Texas, Galveston, Texas, United States

## Abstract

Falls are a leading cause of injury and disability among older adults. This study investigates sex differences in the relationship between grip strength (GS) and cognitive function (CF) with fall risk among Mexican Americans aged ≥75 without history of falls at baseline. Participants (n = 913) were from the Hispanic Established Population for the Epidemiological Study of the Elderly (2004/05-2016). Measures included socio-demographics, body mass index, multi-morbidity, depressive symptoms, and pain. Cognitive function (CF) was assessed using the Mini Mental State Examination (MMSE), with scores < 21 classified as low CF and ≥21 as high CF. Low GS was defined as < 27 kg in males and < 16 kg in females. Participants were categorized into four groups by sex according to GS and CF (High GS-High CF, Low GS-High CF, High GS-Low CF, Low GS-Low CF). Generalized estimating equation models were utilized to estimate the odds ratio (OR) and 95% Confidence Interval (CI) of falls as a function of GS and CF groups. Male participants in the group of high GS-high CF had lower odds (OR = 0.52, 95% CI = 0.30-0.92) of reporting falls over time than those with low GS-Low CF group after controlling for all covariates. No significant association was found between low GS-high CF and high GS-low CF groups with falls. No significant association was found between GS and CF groups among female participants over time after controlling for all covariates. Mexican American older adults who performed high in GS and CF were at decreased odds of reporting falls over time.

